# Is a Meal without Wine Good for Health?

**DOI:** 10.3390/diseases6040105

**Published:** 2018-11-16

**Authors:** Jean-Pierre Rifler

**Affiliations:** Haute Côte d’Or hospital center, F-21400 Châtillon-sur-Seine, France; jprifler@hotmail.com

**Keywords:** wine, Mediterranean diet, Okinawa diet, health, nrf2, alcohol, polyphenols, hormesis, cardiovascular protection, cancer, Alzheimer, metabolic disease

## Abstract

Hippocrates, the father of medicine, had said: “Wine is a thing wonderfully appropriate to man if, in health as in disease, it is administered with appropriate and just measure according to the individual constitution.” Wine has always accompanied humanity, for religion or for health. Christians and Jews need wine for the liturgy. For Plato, wine was an indispensable element in society and the most important in the symposium. In this second part of the banquet, mixed with water, the wine gave the word. If the French paradox made a lot of ink flow; it was the wine that was originally responsible for it. Many researchers have tried to study alcohol and polyphenols in wine, in order to solve the mystery. Beyond its cardiovascular effects, there are also effects on longevity, metabolism, cancer prevention, and neuroprotection, and the list goes on. The purpose of this work is to make an analysis of the current knowledge on the subject. Indeed, if the paradigm of antioxidants is seductive, it is perhaps by their prooxidant effect that the polyphenols act, by an epigenetic process mediated by nrf2. Wine is a preserve of antioxidants for the winter and it is by this property that the wine acts, in an alcoholic solution. A wine without alcohol is pure heresy. Wine is the elixir that by design, over millennials, has acted as a pharmacopeia that enabled man to heal and prosper on the planet. From Alvise Cornaro to Serge Renaud, nutrition was the key to health and longevity, whether the Cretan or Okinawa diet, it is the small dose of alcohol (wine or sake) that allows the bioavailability of polyphenols. Moderate drinking gives a protection for diseases and a longevity potential. In conclusion, let us drink fewer, but drink better, to live older.

## 1. Introduction

The French paradox, a concept described by Serge Renaud, describes the observation that, in France, despite a high consumption of saturated fats, a low cardiovascular mortality rate is described, compared to other “industrialized” countries that consume the same type of food.

The explanation of this French paradox consists of a moderate consumption of wine during meals. There is also a north-south gradient, with an even lower cardiovascular mortality rate in Toulouse (consumption of red wine, olive oil, and duck fat) as compared to Lille, where meals are more based on saturated fats and where the favorite drink is beer.

Although the consumption of red wine is decreasing, the eating habits of adults are changing towards a Mediterranean-style meals and wine remains a social link. The risk of considering wine as just another spirit, is that our young people no longer seek the pleasure of a conversation around a good meal and a good bottle, but look for an immediate euphoric effect, such as in binge-drinking or the holiday heart syndrome. It would be necessary to follow a policy of educating young people so that they turn to wine, rather than a premix or other strong alcohol, to rediscover the pleasure of a reasonable consumption of wine which does not promote addiction. 

We know the protective power of a regular and moderate consumption of red wine, in terms of primary and secondary prevention, and prevention of cancer (particularly studied for resveratrol). We are now interested in its effects on aging and, in particular, its protective role on the occurrence of dementia. 

The ideal dose seems to be two to three standard drinks a day. This is already what St. Benedict recommended for his monks, in his rule number 40 [1]. The proposed amount was one hemin a day, which corresponds to three glasses a day. On the other hand, this did not oblige the monks to abstain, but specified that the fat monks could take a little more. The red wine was, back then, the only source of antioxidants for the winter, as canning and freezing did not exist. It is well established that alcohol, in a glass of wine, improves the bioavailability of polyphenols in the food bolus. This is the principle of the Cretan diet. It is also one of the factors that make the Japanese diet beneficial, in addition to its richness in omega 3 polyphenols and antioxidants (tea catechins, ginger, wasabi, etc.). History has shown that our civilization has always been closely linked to wine. Vinification allows the extraction and the conservation of the antioxidants, thanks to the alcohol, and it is still the wine’s alcohol that allows our body to absorb the antioxidant polyphenols that are beneficial to our health [2]. Why demonize this product, which has long been one of the only effective products of our former pharmacopoeia?

## 2. Free Radicals and Antioxidant Defense 

Reactive Oxygen Species (ROS) can be exogenous and endogenous. Exposure to pollution, prolonged sunlight, absorption of drugs, alcohol, and smoking causes ROS production that can outperform the endogenous antioxidant defenses. Unfortunately, there are not enough fruits and vegetables in the food consumed by people (polyphenols, vitamin C, vitamin E, and carotenoids) that can boost the endogenous antioxidant defenses.

**Figure 1:**
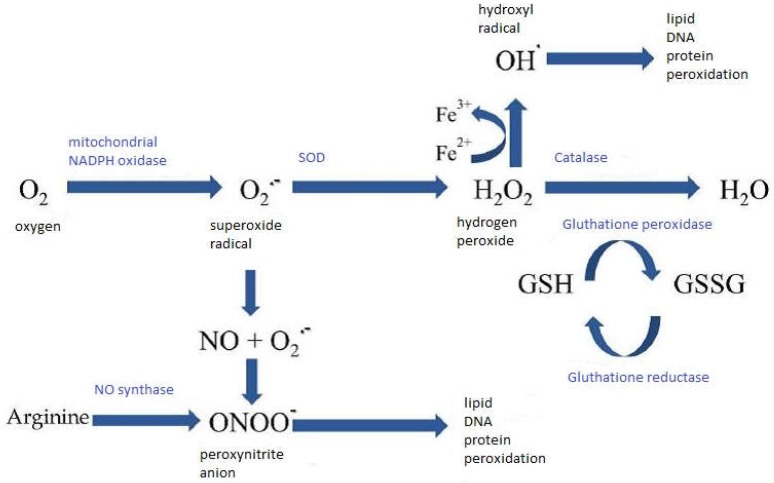
Production of Reactive Oxygen Species.

All cells, by their metabolism, produce small amounts of derivative oxygen reagents. Indeed 1 to 2% of the oxygen present is diverted to form free radicals. ROS are mainly produced at the mitochondrial level during the process of conversion of oxygen into water, producing a superoxide radical (O^2−^). This superoxide radical can also be produced at the microsomal and plasma levels, by Nicotinamide Adenine Dinucleotide Phosphate (NADPH) oxidases. The superoxide radical is converted into hydrogen peroxide (H_2_O_2_) (more stable) by Superoxide Dismutase (SOD), and then into water, either by catalase (CAT) or glutathione peroxidase (GPx) [3]. ROS can react with different biological substrates, such as lipids, proteins, and DNA. Oxidative stress is the balance–imbalance of prooxidant and antioxidant compounds. ROS are involved in the expression and regulation of the functions of cell proliferation and cell death. The study of various pathologies, such as neurodegenerative diseases, atherosclerosis, and cancer, has shown that ROS also act as inflammatory mediators. ROS are very unstable, and their lives are very short (10^−4^ seconds). Their reactivity lies in their search for an electron to match their single electron. O_2_•^−^ and hydrogen peroxide (H_2_O_2_) are not very active. O_2_•^−^ can capture H^+^ to give HO_2_•, which would be the reactive form of O_2_•^−^, that is capable of initiating a lipid peroxidation. O_2_•^−^ can also be dismuted to H_2_O_2_ and O_2_ (spontaneous reaction or catalyzed by superoxide dismutase), react with NO• to form the peroxynitrite anion ONOO^−^, a powerful oxidant, or reduce the transition metal ions. O_2_ is produced, in particular, by the reduction of molecular oxygen in mitochondria by NADPH oxidase or by xanthine oxidase, an enzyme of the purine metabolism. The hydroxyl radical HO• is one of the most oxidizing chemical species and can very quickly attack most biological molecules. HO• is produced by the reduction of H_2_O_2_ by low-valence metal ions, such as Fe^2+^ or Cu^2+^, free or complexed (heme); the Fenton reaction (Figure 1).

Mitochondria are the essential organelles responsible for the production of energy, in the form of ATP, which is necessary for the cell function. The respiratory chain is a permanent source of ROS. Complexes I and III are the preferred sites for the ROS production [4,5,6].

Expression of nuclear genes encoding mitochondrial proteins, as well as mtDNA replication and transcription mechanisms, are regulated primarily by transcription factors and transcriptional coactivators. 

### 2.1. Transcriptional Factors Nrf2 (Nuclear Respiratory Factor 2)

Nrf2 is an important transcription factor that protects the mitochondria from stress oxidants by inducing anti-oxidant and detoxification genes, by its binding to the Antioxidant Response (ARE). However, it would have a facilitating role on the formation of atheroma. The Keap1 protein binds to the Nrf2 protein to inhibit it. In the quiescent state, Nrf2 is anchored in the cytoplasm by binding to Kelp1 protein (Kelch-like ECH-associated protein 1), which facilitates the ubiquitination and proteolysis of Nrf2. This mechanism contributes to the repressor effect of Keap1 on Nrf2. Activation of Nrf2 leads to a coordinated antioxidant and anti-inflammatory response [7,8,9,10,11,12]. 

### 2.2. Antioxidant Defenses

#### 2.2.1. Enzymatic Defenses Systems

Enzymatic systems are the most important cellular defense systems to control oxidative attacks. They protect or detoxify the body against ROS. 

Superoxide dismutases (SOD) and catalase (CAT) play a protective role, while glutathione peroxidases (GPx) play a role in detoxifying the ROS. There are three SODs: That located in the cytoplasm involving copper and zinc as co-factors, named Cu^2+^/Zn^2+^-SOD (SOD-1); that located in the mitochondria which has manganese as a co-factor, Mn^2+^-SOD (SOD-2); and the secreted extracellular Cu^2+^/Zn^2+^-SOD (SOD-3). SODs are metalloenzymes that catalyze the disproportionation of superoxide anions into hydrogen peroxide and oxygen, ten thousand times faster than the spontaneous disproportionation of the superoxide anion. The reduction of H_2_O_2_ in the cytosol will depend on the Cu^2+^/Zn^2+^-SOD and on glutathione peroxidase (GPx). Catalase is localized in the mitochondria and peroxisomes, it can also reduce H_2_O_2_, but due to its low affinity for this radical, GPx is more efficient [13]. On the other hand, an excess of H_2_O_2_ or the presence of transition metals, Fe^2+^ and Cu^2+^, can lead to the formation of the hydroxyl radical (OH) and nitro reactive metabolites. GPx detoxify hydrogen peroxide and lipid peroxides by coupling their reduction to oxidation of a reducing substrate, glutathione [14]. GPx are seleno-dependent enzymes that contain four selenium atoms in the active center of the enzyme. Thus, a selenium deficiency causes a decrease in the GPx activity, while an abundance restores it. Two forms of GPx differ from each other in structure and activity [15,16].

#### 2.2.2. Non-Enzymatic Antioxidant Systems

Non-enzymatic antioxidants may be endogenous water-soluble agents (glutathione, uric acid, bilirubin, and ubiquinol (coenzyme Q10)) or be provided by the diet (vitamin C and E, carotenoids, polyphenols) [17,18,19]. Glutathione (GSH) is an endogenous water-soluble agent that has important antioxidant properties. Glutathione prevents the oxidation of thiol groups, thanks to its reducing power. The regeneration of the GSH thiol function from the oxidized form is done through the activity of the glutathione reductase (GR). It can chelate the cuprous ions and, thus, limit their participation in the Fenton reaction. In addition, it is directly involved in the repair of the oxidative damage to DNA. Water soluble vitamin C can behave as an antioxidant or prooxidant, depending on the dose used. Too high a dose of ascorbic acid becomes toxic to the body. Ascorbic acid can take a reduced or oxidized form, depending on the pH of the medium in which it is and the presence of transition metals. The passage from one form to another is affected by glutathione/glutathione reductase and it generates an ascorbyl radical. Thus, it is considered as a redox couple with an ascorbyl radical, capable of capturing the ROS and the singlet oxygen. Vitamin E exists in eight natural forms, four tocopherols and four tocotrienols. Tocopherol is liposoluble and has the capacity to capture and stabilize the single electron of free radicals, following the reaction:

tocopherol-OH + LOO • −˃ tocopherol-O • + LOOH (LOO •: free radical lipid)

The radical-bearing tocopherol may react with a new free radical to form a neutral species, or be regenerated by vitamin C, glutathione, or the coenzyme Q10. Vitamin E mainly plays its role as an antioxidant in biological membranes. Mitochondria, which generates free radicals, contain high levels of vitamin E in their lipid membrane, consisting of polyunsaturated fatty acids and are subjected to oxidative stress.

**Figure 2:**
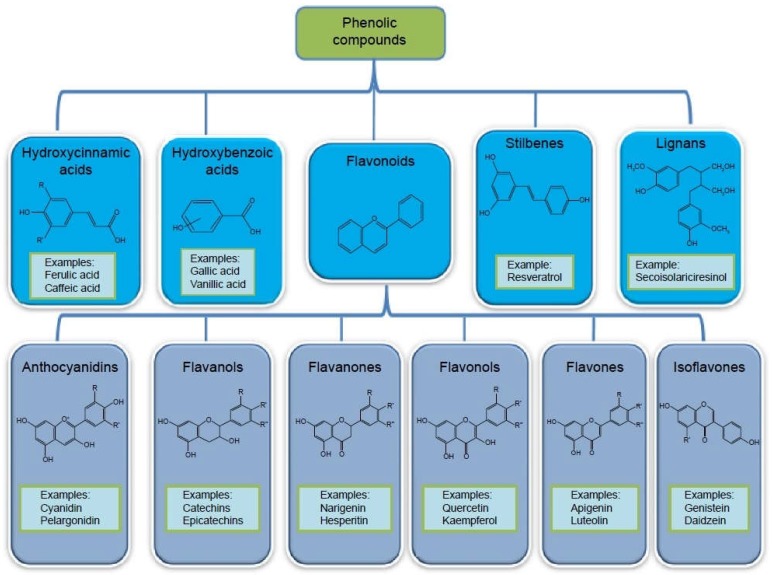
Phenolic Compounds.

Natural polyphenols include a broad set of chemical substances, comprising at least one aromatic nucleus, bearing one or more hydroxyl groups, in addition to other components. They can range from simple molecules, such as phenolic acids (gallic acid), to highly polymerized compounds of more than thirty thousand daltons, such as tannins (tannic acid). Polyphenols are commonly subdivided into simple phenols, phenolic acids (derived from benzoic or cinnamic acid), stilbenoids (two C6 rings linked by two carbon atoms), flavonoids, isoflavonoids and anthocyanins, and condensed tannins (Figure 2).

**Figure 3:**
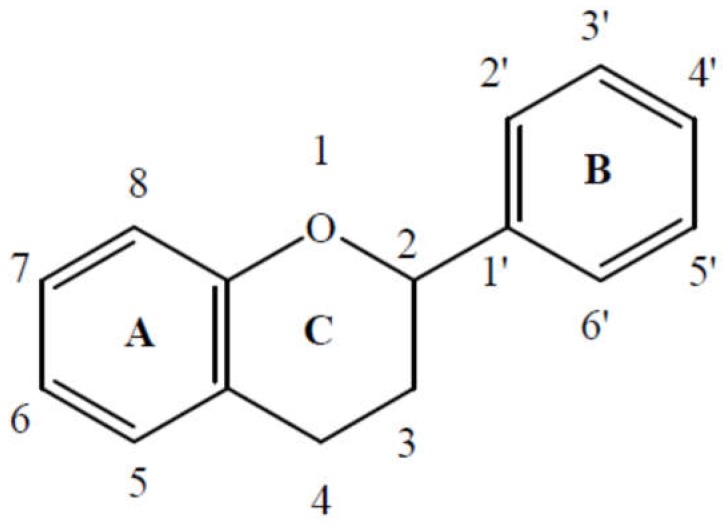
Flavonoids structure.

The fruits and vegetables consumed provide more than eight thousand polyphenols. Flavonoids are the most abundant polyphenols in our diet and over four thousand have been identified [20]. Flavonoids have a common C6-C3-C6 structure. Two aromatic rings (A and B) are linked by a chain of three carbons forming an oxygenated heterocycle (C) [21,22] (Figure 3).

Among the flavonoids, flavanones are responsible for the bitterness of grapefruit. The tannins are responsible for the astringency of various fruits (skin and grape seeds) and anthocyanins, the color of red fruits. Polyphenols are present in various natural substances, in the form of anthocyanin in red berries and red wine, as proanthocyanidines in chocolate and wine, as coffee-quinoline and feruloylquinic acids in coffee, flavonoids in citrus fruit, and in the form of catechins, such as epigallocatechin gallate in green tea, quercetin in apples, onions, and red wine. These basic carbon skeletons are derived from the secondary metabolism of plants, developed by the shikimate pathway [23]. Shikimic acid is an important biochemical intermediate in plants and microorganisms. It was isolated for the first time in 1885 by the Dutchman Johann Frederik Eijkmann, from the flower of shikimi (illicium anisatum or Japanese badian). Shikimic acid is the precursor of phenylalanine and tyrosine, aromatic amino acids; indole, indole derivatives and tryptophan; many alkaloids and other aromatic metabolites, such as resveratrol; tannins; lignin; and salicylic acid.

**Figure 4:**
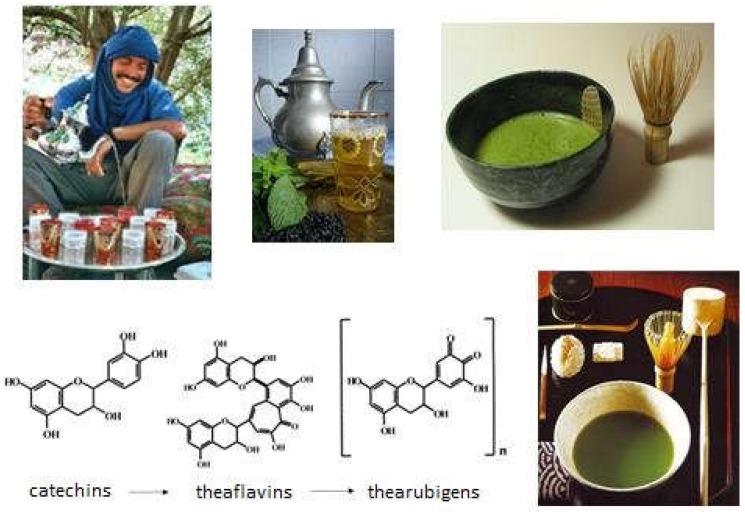
Polymerization of catechins.

Polyphenols in wine are present in the film and seed. In the roundup, tannins are often undesirable, from a taste point of view, and maceration is most often performed on the broken-up grapes. The solubilization of these polyphenols takes place during the alcoholic fermentation, it increases with the alcoholic degree of the grape must. Maceration is the stage of winemaking that extracts the phenolic compounds. The condensed tannins are oligomers or polymers of flavanols. They consist of flavan-3-ols units, linked together by carbon-carbon bonds. The conformations are helicoidal. The passage in barrels (breeding) allows a micro oxygenation of the wine, through the pores of the wood, and the polymerization of the tannins. The barrel provides little or no wood polyphenols. It is this polymerization of tannins by microoxidation, which confers on them their anti-oxidant properties. This same phenomenon is used for tea—microoxidation by successive passages of the tea through glass teapots, among the Arabs, or microoxidation with the tea whip macha (chasen), among the Japanese (Figure 4).

## 3. Wine and Cardiovascular Protection: The French Paradox

Many studies have tried to unravel the “French Paradox”, a term used by Serge Renaud in 1991 on CBS. This paradox consists of a low mortality of cardiovascular origin, despite a high consumption of saturated fats [13,24]. The epidemiological studies of Saint Leger [25] and Keys [14] (thirteen thousand subjects followed for twenty years, beginning in 1952) showed that the Mediterranean basin, and more particularly Crete, was protected, probably because of a specific diet. The MONICA project [26] confirmed the particular position of France, and showed a south-north mortality gradient, confirming the probable origin of the difference in diet between Toulouse and Lille. The intake of mono and polyunsaturated fats, garlic, duck fat, low-meat diet, high intake of fruits and vegetables, have been advanced, to identify the Mediterranean diet; the Cretan diet is also characterized by this frugality. All Mediterranean civilizations are also wine civilizations, and some wanted to study an important or even essential element of this diet that is protective against cardiovascular diseases. The antioxidant effect of red wine flavonoids appears to be one of the mechanisms of vascular protection provided by the Mediterranean diet. The term “French paradox” describes the paradoxical situation in which the French population, more particularly the southern one, dies less of cardiovascular pathology than northern Europeans and Americans, despite high and comparable risk factors (smoking, rich diet including saturated fats, sedentary lifestyle). Given the lack of a fundamental difference in the diets of the different populations studied, Serge Renaud spoke of the role of red wine consumption, because the French consume 89 liters of red wine a year against 7.7 for the British [27]. The French paradox was then attributed to the consumption of wine because the French are the largest consumers of wine in the world (after the Luxembourgers) [28]. French gastronomy is reputed to be rich in lipids, especially in saturated fatty acids, with more than four hundred cheeses, charcuterie, mayonnaise, butter, foie gras, etc., in which it does not differ much from American or British food. The Mediterranean-type diet only concerns the South of our territory, that is to say 20% of the French [29]. The French, compared to other Western countries, rarely eat alone and prefer conviviality (work colleagues, family, friends, and neighbors).

If, as in all developed countries, the French mostly frequent supermarkets, they also do not neglect the small shops (bakeries, fishmongers, butchers, grocers, and markets) thus, favoring quality over quantity, in contrast to English people who are more likely to buy their groceries in bulk, during one visit to a supermarket, which already suggests more energy spent on supplies [30]. The French take more time to cook their dishes. They take more time to eat without consuming more. A discussion usually animates the meal. The gourmand/gourmet distinction is not so clear among the Anglo-Saxons. “Being gourmet” is more valued socially in France, than elsewhere. According to the American Time Use Survey (ATUS) [31], the average length of a meal in France is 15 minutes at breakfast, 38 minutes at noon, and 40 minutes at night, for a total of 93 minutes per day at the table, which is 30 minutes longer than the time spent by the Americans. The prolonged average duration of meals has a beneficial effect on the absorption and metabolism of fats, as well as on the peak-level of insulin secretion. The French eat less, between meals, to better appreciate the quality of their cooking (7.5% of our daily calories against 21% for Americans). Contemporary reality shows, such as “top chef” or “almost perfect dinner” add value to our national culinary expertise, on a daily basis. Two-thirds of French people eat at home at noon [32] and do not consume frequent snacks—only 6% consume fast food more than once a week [33]. We eat less red meat, and more cheese and yogurt than whole milk. The Southern French consume more olive oil while those in the North prefer butter. Only 10.2% of French people consume the five fruits and vegetables recommended by WHO. Thirty percent of the French people garden and grow a vegetable garden, which is unusual in Northern Europe. Vegetables are rather consumed fresh, not packaged industrially, and are often raw. The French is known to be a great consumer of garlic and snails. Seventy percent of the French people consume coffee every day. Ruidavets showed in 2004 that wine consumption in France has a positive impact on the quality of diet, compared to that of abstainers [34].

**Figure 5:**
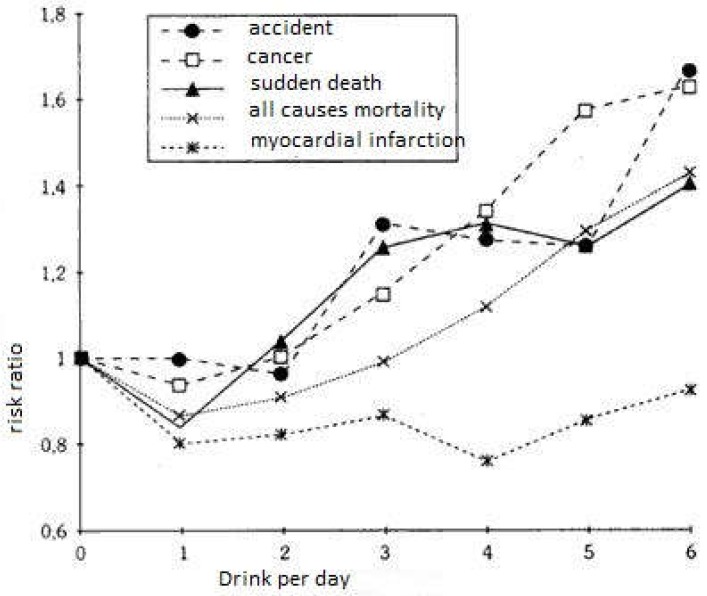
J curve [40].

Generally, the midday meals are varied with successively an appetizer, a main meal, a cheese and then a dessert. The French eat smaller shares than Americans [35], which may already partly explain the French paradox. Montaigne was already talking about French alcohol consumption [36]. It is daily, moderate, and during meals, and conviviality is the most important. It differs from that of other Western countries. In France, drunkenness is a consequence, rarely a goal (except for alcoholics) it is the spreading of a pleasure and not the necessary cause of an intention of overflow. This mode of consumption is beneficial in many ways. Wine is also associated with relaxation, with the pleasure of conviviality. We know from Yusuf’s meta-analysis [37] that moderate alcohol consumption (two drinks a day) is a cardiovascular protective factor, just like that of decrease in LDL, cessation of smoking, consumption of fruits and vegetables, and engagement in physical activity. Atheroma plaque in the coronary arteries is responsible for myocardial infarction and in the carotid artery it causes an ischemic stroke. The mechanism of plaque formation is complex and multifactorial, but begins with an accumulation of oxidized LDL in the intima of the artery. The effect of red wine on the oxidation of LDL could be the key to the French Paradox. Indeed, the decrease of LDL (bad cholesterol) and an increase in the antioxidant power of the serum are two major protective factors. If there is less LDL and they are less oxidized, they will be deposited less in the arteries, so the formation of atheroma plaque will be delayed. The InterStroke study [38] confirms this data for stroke, at doses of one drink per day. Some will make the short cut by saying that a glass for the brain and two for the heart, which makes the three glasses allowed per day, since Saint Benedict. However, before recommending red wine as a beneficial dietary adjunct, it should be noted that the dose of alcohol must remain minimal so as not to cause the known deleterious effects of ethanol. On the other hand, it is certain that, whatever the red wine, it is a soup of antioxidants that can be preserved for a very long time when vinification is carried out in a traditional way (control of temperatures, long maceration, aging in new barrels), which is easily assimilated by the body because of the alcohol [39]. Alcohol provides superior bioavailability. It is not by chance that wine has always been part of the diet of the Mediterranean countries, it was the only way to conserve antioxidants (plants) for the winter. At a time of globalization, canning and freezing, we still have the fact that a glass of red wine brings much more antioxidants easily assimilated than large quantities of fruits and vegetables. The J curve represents the total mortality related to the number of glasses of wine, drunk per day. In Ellison’s version [40] (Figure 5), the ischemic heart disease is isolated from other causes of mortality, if we notice an increase in deaths from accidents, cancers, or sudden death, for a daily consumption of more than three glasses, we notice a cardiovascular protection activity beyond these doses. 

**Figure 6:**
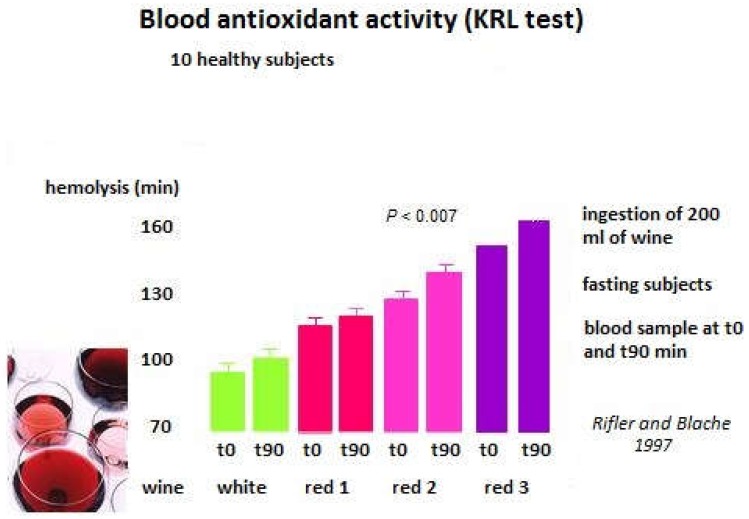
Blood antioxidant activity in healthy subjects drinking wine.

A study published in 1997, showed that regular consumption of moderate doses of wine (200 mL) allows an antioxidant protection (Figure 6). All subjects were in good health (7 men and 3 women). The result was not significantly effective for all the wines. Only one wine had a speed effect on redox blood status (red 2). However, we can see that the antioxidant status was higher after the last wine. Therefore, we can suspect that the regularity of ingestion is the secret for a healthy consumption [41]. All of the tested wines were great wines of bordeaux and burgundy (red 2 was a bottle of Echezeaux of Domaine de la Romanee conti).

**Figure 7:**
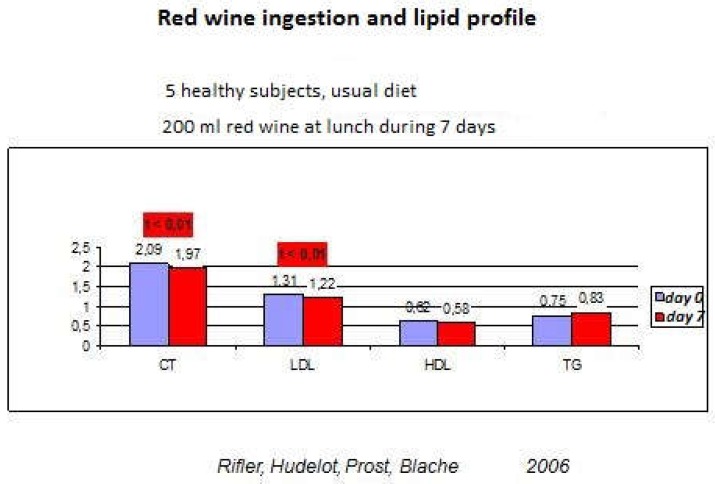
Lipid profile after 200 mL red wine ingestion at lunch. CT: Total cholesterol. LDL: Low density lipoprotein. HDL: High density lipoprotein. TG: Triacyl glycerol.

In another 2006 study, a supplement of 200 mL of red wine, during lunch, of healthy volunteers, showed a decrease in Total cholesterol and LDL cholesterol in just one week [41] (Figure 7). This regular and moderate consumption of red wine makes it possible to counteract the initiating factors of the atheromatous plaque, and have a cardiac protective effect in the primary prevention.

**Figure 8:**
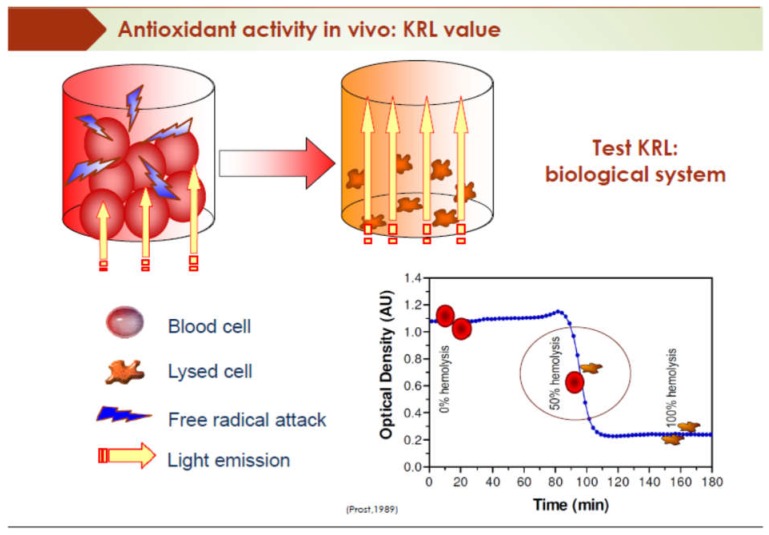
KRL test.

In the secondary prevention, we demonstrated in 2012 [42,43], the same phenomenon on patients with myocardial infarction, three days after the acute accident, a healthy diet was set up, with a drinking group of water, and the others drinking red wine (2 glasses a day). The wine group has demonstrated the same benefits as the primary prevention. The aim of this study was to study the effect of moderate and regular wine consumption on relevant biological parameters (lipid balance, measurement of serum total antiradical resistance, level of fluidity of the red blood cell membrane) in patients in a situation of secondary prevention of a cardiovascular event, during their hospitalization for cardiovascular rehabilitation. Two similar populations were distinguished only by the consumption of wine. Thus, wine was the only hygieno-dietary parameter differentiating the two groups. The dietary diet chosen “Western prudent”, was based on the dietary principles of the Lyon study, which is the reference in terms of cardiovascular protection, because of its resemblance to the Mediterranean diet. It consisted of a limited intake of saturated fats (animal fats, oils), a daily intake of fruits and vegetables, butter is replaced by olive oil and rapeseed, and limitation of cheese intake (2–3 times a week). The wine was made from the Pinot Noir grape variety, Villars Fontaine Haute Côte de Nuit, vintage 1999, with a high content of phenolic compounds (around 4000 mg/L of gallic acid equivalent). The wine was served without a label and without other indications. The consumption of wine was done exclusively during meals (1 glass at lunch, 1 glass at dinner). The anti-radical defense system is so complex that no specific test can provide an overall assessment of an individual’s resistance to the attack of free radicals. The KRL^®^ test used (Figure 8), was initially developed by the Spiral/Kirial International laboratory, at the request of NASA in 1987 [44]; it is a global measure of the antiradical defense potential. The KRL test makes it possible to evaluate the overall resistance against the aggression of free radicals. Thus, it is hoped that the higher the level of KRL, the less the individual will subject his arteries to the process of atherogenesis.

The blood sample is subjected to a standardized and reproducible aggression, induced by a generator of free radicals. The enzymatic and chemical defense systems allowing the integrity of the red blood cells are tested until cell lysis is obtained. The time after which 50% of the blood cells are lysed (half-hemolysis time, t_1/2_ in minutes), thus, reflects the overall antioxidant potential KRL 

The results between the group of drinking subjects versus the group of abstinent subjects showed an increase in the HDL level, a decrease in the LDL level, an increase in the antioxidant potential, an increase in the fluidity of the erythrocyte membrane. The results in the group of drinking subjects between D0 and D14 showed a decrease in cholesterol and LDL, an increase in the antioxidant potential of the serum, an increase in the fluidity of the erythrocyte membrane, and an increase in the VO_2_ max (maximum oxygen consumption) of 26%. A decrease in LDL and an increase in antioxidant serum are two protective factors. If there is less LDL and they are less oxidized, they will be deposited less in the arteries, so the formation of atheroma plaque will be delayed. It is possible to hope that the significant antioxidant effect obtained as early as D15, as well as the decrease in LDL levels, will have a retarding effect on the progression of the atheroma plaque. Similarly, the increase in membrane fluidity allows red blood cells to circulate better through an atheromatous artery.

### 3.1. Self-Action of Ethanol

High Density Lipoprotein rate increase: The oldest known mechanism is the increase in high density lipoprotein (HDL) levels [45,46,47], including the HDL2 and HDL3 fractions, as well as the protein constituents of HDL, Apo A1, and Apo A2 [48]. A decrease in the activity of the cholesterol transfer protein esterified, CETP [49] could be the origin of the rise of HDL. Prospective studies have found an inverse correlation between HDL levels or its fractions and the risk of ischemic heart disease [50]. Indeed, HDL is a protective factor against the formation of atheroma due to a transport of cholesterol from the vascular walls into the liver, with a biliary elimination. An increase in the HDL-linked enzyme paraoxonase protects against the LDL oxidation [51], without this increase depending on the type of alcoholic beverage. On the other hand, at high doses, ethanol increases the triglyceride level and remains neutral on the LDL level.

A decrease in platelet aggregation: Platelet aggregation is decreased by low or moderate doses of alcohol [52,53]. However, after heavy ingestion of alcohol, a rebound effect on the platelet response can be observed [54], causing sudden death. 

A decrease in fibrinogen: An increase in blood fibrinogen is considered a risk factor for ischemic cardiovascular disease. According to Ridker’s study [55], the concentration of fibrinogen correlates positively with alcohol consumption, with a U-shaped curve (higher concentrations for non-drinkers and those who drink more than 60 g/day alcohol). This association was valid for wine and spirits, but not for beer and cider. A low level of fibrinogen in moderate-drinkers may explain the reduction of ischemic heart diseases in the latter.

Thrombolysis: Stimulation of plasminogen activator has been observed [56]. 

Tissue factor: A reduction of this protein, which probably plays an important role in atherogenesis, has been reported in regular drinkers [57]. 

Homocysteinemia: Plasma elevation of this amino acid is considered a risk factor for cardiovascular disease. Some studies have shown a decrease in plasma levels of homocysteine in very low or moderate alcohol users [58]. However, several studies show an increase in plasma homocysteine, occurring during the consumption of alcoholic beverages, regardless of the amount of alcohol consumed [59], which goes against the previous results. 

Effects on blood pressure: Substantial alcohol consumption is accompanied by a rise in blood pressure [60] but for the doses we are interested in here, the effect is rather opposite, through arterial vasodilatation.

### 3.2. Self-Action of Polyphenols

Phenolic compounds are classified into cinnamic acids, benzoic acids, stilbenes, lignans and flavonoids (themselves classified as anthocyanidins, flavanols, flavones, flavonols, and isoflavones) [61]. Epidemiological studies have correlated the consumption of plant polyphenols with a low incidence of coronary heart disease. The polyphenols in red wine have antioxidant and free radical scavenging properties. They protect LDL against oxidation. These polyphenols decrease platelet aggregation and inhibit the proliferation of vascular smooth muscle cells. Polyphenols stimulate the production of relaxing factors of vascular endothelium, such as nitric oxide. Finally, they contribute to preserving the integrity of the vascular endothelium by acting on both proliferation, migration, and apoptosis of endothelial cells. All these properties confer on the polyphenols the possibility of interfering with the atherogenic process or the thrombotic phenomena associated with atherosclerosis, and could explain the vasculo- and cardio-protective effects of these compounds [62] Since wine-induced cardiovascular protection appeared to be superior to that of other alcoholic beverages, many authors have sought to demonstrate that this was a consequence of the wine’s high antioxidant content [63,64,65]. Indeed, the red wine contains seven to ten times more tannins than the white wine, and according to the grape varieties there are different levels of polyphenols. Resveratrol and flavonoids are the two most-studied molecules today.

Reduction of LDL oxidation: In vitro, the fact that antioxidants (vitamin E, carotenoids, polyphenols, vitamin C, etc.) slow the oxidation of LDL induced by Cu^2+^ when they have been absorbed at high doses by humans, has been demonstrated many times [66,67]. Fuhrman [68] showed that subjects consuming red wine had LDL that was more resistant to oxidation. Kanner’s team [69] shows that during a high-fat meal (red meat and fries), the absorption rate of malondialdehyde, an oxidative substance produced during triglyceride degradation and responsible for peroxidation LDL in the blood, was reduced by 75%, if there is consumption of red wine (or green tea), during the meal! This rate goes to 0% absorption if the meat was marinated in wine, before cooking. We have here an attractive explanation of the French paradox. These researchers describe the stomach as a bio-reactor in which the wine prevents the oxidation of fats. This study confirms the recommendation of drinking during meals. Blache, shows that oxidized HDL lose much of their property in removing cellular cholesterol [70]. He concludes that HDL, like LDL, are sensitive to oxidation and that antioxidants can provide protection by preserving their functional abilities. Resveratrol lowers the triglyceride levels [71].

Action on platelet aggregation: This was Renaud’s initial hypothesis [72] to explain the French paradox. Resveratrol is also capable of inhibiting platelet aggregation [73] by inhibiting the production of cyclooxygenase, resulting in a decreased thromboxane formation [74,75]. The effects are close to those obtained with aspirin. In a study conducted at the University of Burgundy, it was shown that a wine rich in resveratrol was more effective on the parameters of atherothrombogenesis than a wine poor in resveratrol, in volunteers who ingested three glasses per day, during two weeks. Similar results have been demonstrated in vitro, with flavonoids such as quercetin [76]. 

Atherogenesis: The antioxidant action of polyphenols appears to be targeted on LDL, as recalled by the classically accepted physiopathology of atheromatous plaque. If paradoxically, the oxygen we breathe has a vital role for our survival, its oxygenated derivatives, free radicals, are the cause of the multiple phenomena that underlie cellular aging in general, pathologies and atherogenesis, in particular. Many factors and cells intervene in this mechanism, not all of them are clear. The vasodilator effect of NO, whose secretion is mediated by polyphenols via the NO-syntase, is one of them. [76]. New data on the mode of action of polyphenols is challenging the paradigm of antioxidant flavonoids and vasodilator alcohol. Indeed, the bioavailability of these molecules is so low that their mode of action as a direct antioxidant scavenger, is unlikely, in the blood [42].

## 4. Polyphenols and Cancer

Polyphenols intervene, partly, by action on the estrogen receptors. For example, tea polyphenols have actions on the different stages of cancer development by blocking initiation, promotion, and progression [77]. Many studies show a modulatory effect of flavonoids on the mechanisms of apoptosis [78]. Chalopin, in 2010, discovered that polyphenols could act via the estrogen receptors [79,80]. This explains the relative protection of women, during periods of genital activity. If the process by which the isoflavones of foods interact with breast cancer cells is unclear, the research points to antioxidant, anti-inflammatory, anti-angiogenic effects that, therefore, influence the survival and growth of the tumor. It is a demonstration of the influence of certain lifestyle factors, including nutrition, on cancer survival, after diagnosis. Survival is better in patients who consume more natural dietary isoflavones (and not isoflavone supplements) [81]. Flavonoids also exert an anti-aromatase action and may reduce the proliferative effect of estrogens, in this way, in the case of hormone-dependent cancers [82]. While the protective effect is fully demonstrated for Asian populations consuming soy isoflavones since childhood, the effect on European populations has not been demonstrated, but there is a possible evidence to support efficiency. In fact, moderate consumption of wine seems to be beneficial for both men and women. One of action seems to be via the estrogen receptors, which could explain the positive effect outside of hormone-dependent breast cancer [83]. A particular point on resveratrol, this stilbene, which has been the most studied. Its anti-cancer effect in vitro has been studied by many teams, with a protective action in the three stages of carcinogenesis—initiation, promotion, and progression [84]. Resveratrol has a multi-organ action, it acts on inflammation and free radicals, it is immunosuppressive, antitumoral, and its actions go through signaling pathways, estrogen receptors, and sirtuins [85].

## 5. Polyphenols and Metabolism

Foxo1 is a transcription factor of the Forkhead box family (FOXO) that regulates various signaling pathways, including oxidative stress, programmed cell death, catabolism, and insulin sensitivity [86]. The transcription factor Foxo1 is a key player in insulin transcriptional responses and plays a central role in metabolic adaptation during fasting. The major role of Foxo1 and its counterparts is protection against oxidative stress and DNA damage, and thus in determining longevity. The binding of insulin to its receptor triggers a series of phosphorylation—in the order IRS, PI3K, Akt, and Foxo1. Phosphorylated Foxo1 can no longer migrate to the nucleus to induce transcriptional activation. The Sir2 (silencing information regulator) gene is capable of increasing the number of divisions of the same cell by, approximately, 30%. Mammals have 7 Sir2 homologs called sirtuins (SIRT1-7), and it is the SIRT1 protein that appears to be functionally closest to sir2. SIRT1 “silent information regulator 1”, is a NAD-dependent deacetylase, whose activity, therefore, directly depends on the nutritional state since it varies the NAD/NADH ratio. The longevity caused by caloric restriction, a condition that increases the NAD/NADH ratio, would at least, partly, pass through Sir2/SIRT1. It is involved in various processes [87,88]—inflammation, energy restriction, mitochondria biogenesis, stress resistance, cellular senescence, endothelial function, apoptosis, and circadian rhythm. It decreases transcription of the P53 protein, which decreases apoptosis [89]. The E2F1 protein stimulates the expression of sirtuin 1, which in turn inhibits the activity of the first and its apoptotic activity. It is also, by this means, protective against DNA damage [90]. By combining with a FOXO protein, it protects the cell against oxidative stress [91]. In mice, with a mutation in the SIRT1 gene resulting in the production of a non-functional protein, lethality is important in utero, with surviving individuals showing multiple tumors, probably secondary to an inability to repair DNA. [92]. SIRT1 can, therefore, be considered a tumor suppressor gene.

The effect of resveratrol on sirtuins would produce an effect mimicking caloric restriction and increasing longevity, a beneficial effect on type 2 diabetes also seems possible, by increasing the insulin sensitivity and lowering the blood glucose [93,94]. Low wine consumption would increase life expectancy by five years [95]. It has long been shown that caloric restriction is in itself one of the criteria for extending life expectancy, universally [96,97]. The principle of caloric restriction is to leave the table without having access to the sensation of satiety or by accessing it by a high-consumption of foods with a low-caloric index.

The Cretan diet is the combination of a frugal diet, low calories (caloric restriction), and the Mediterranean diet type. Explaining the excellent life expectancy of the Cretans. 

Fifteen percent of the world’s super-centenarians (over 107 years old) live on the island of Okinawa. The Okinawa diet is the application, with a Japanese diet, of an organized calorie restriction:

- Stop eating before being completely satiated (Hara Hachi Bu).
- Eat only small portions (kuten gwa).
- Eat with the thought that food has healing powers (nuchi gusui).
- Promote a variety of foods.
- Eat fresh foods.
- Combine raw and cooked foods.
- Cook little food over low heat.
- Avoid the microwave oven and barbecue.
- Give preference to colors on the plate.

This action of polyphenols on sirtuins is the key to protecting against diabetes and increasing the longevity of moderate wine drinkers.

## 6. Polyphenols and Alzheimer

Alzheimer’s disease is an incurable neurodegenerative disease of brain tissue that results in the progressive and irreversible loss of mental functions, including memory. It is the most common cause of dementia in humans. It was initially described by the German doctor Alois Alzheimer in 1906. Two types of nerve tissue damage characterize Alzheimer’s disease: Senile plaques (or amyloid deposits) and neurofibrillary degeneration. The constituents of these lesions are, respectively, the amyloid peptide (or Aβ) and the Tau protein. The positive effect of wine on neurodegeneration has been confirmed by numerous studies, confirming that alcohol has a more general vascular action. The PAQUID (Personnes Agees QUID) study [98,99] identified the main neuroprotective factors, of which wine is a part. Here again, we fear an association bias between wine consumption and socio-economic levels, with favorable dietary rules. Over thirteen years of study, in patients aged over 65 years in the Gironde and Dordogne, classified as non-drinkers, light-drinkers, moderate-drinkers and heavy-drinkers, it appeared that there was no difference between the non-drinkers and the light-drinkers, for risk of Alzheimer’s disease. It is from three or four glasses a day that the benefit appears and increases with time. This study showed a lower decline in cognitive function in subjects drinking moderately, while it was generally agreed that even at very low doses, alcohol consumption could induce cognitive impairment. A study conducted in Rotterdam [100], on 5400 subjects over 55 years, concluded that vascular dementia is also less common among drinkers, whether beer or wine. A study also confirms this in women [51,101].

Moderate alcohol consumption improves the mood and quality of life of older men and women [102], by promoting better sociability. It also helps stimulate appetite. Resveratrol decreases the secretion of beta amyloid protein in rats [103]. The Bordeaux study of Orgogozo and Dartigues [99] showed a significant decrease in dementia and Alzheimer’s disease for consumption of 4 to 5 glasses of red wine, per day.

## 7. Hormesis

**Figure 9:**
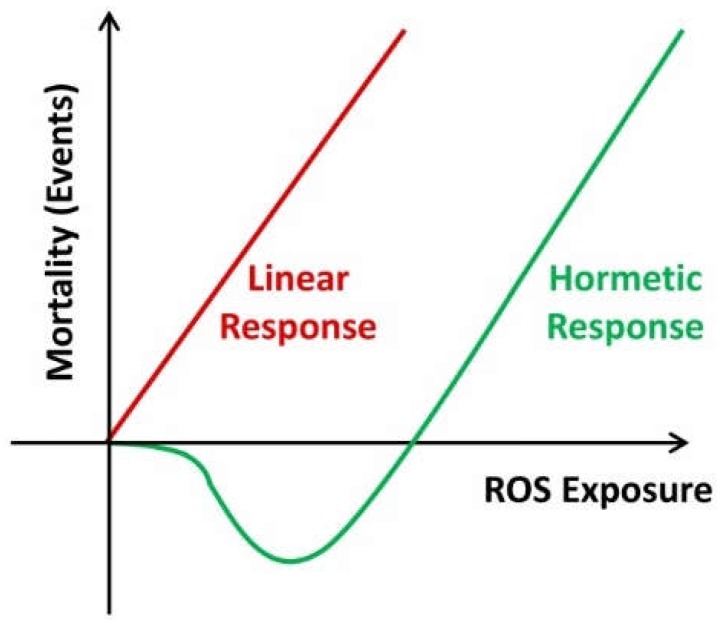
Hormetic response.

Hormesis designates a response of stimulation of the biological defenses, generally favorable, with exposures of low doses of toxins or other agents or phenomena stress generators. As a result of this mechanism, some natural toxins or pollutants may have the opposite effect, depending on whether the dose received is low or high. These agents are said to be hormetic. In toxicology, the phenomenon of hormesis is characterized by a characteristic form of the dose/effect relationship curve, which changes sign for low doses, giving it a “U” or “J” shape (Figure 9).

It is usually accepted as a dose-dependent response to a stimulus. The current hypothesis is rather a response to a prooxidant background mediated by nrf2 (nuclear respiratory factor 2) [103]. Normally, moderate oxidative stress will favor the nrf2 pathway responsible for the endogenous synthesis of anti-oxidant enzymes. In case of overflow, it is the inflammatory NFkb pathway that will be triggered with a passage to apoptosis [104]. 

**Figure 10:**
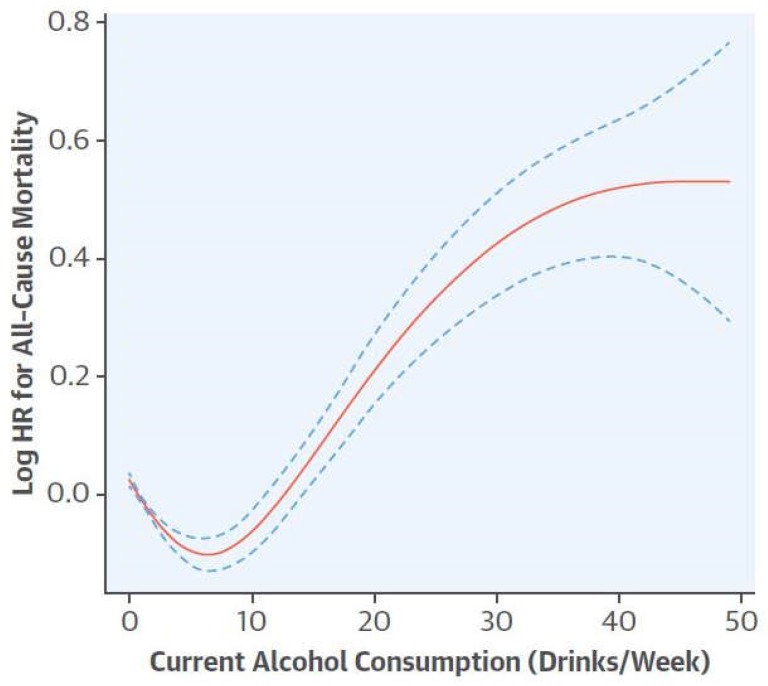
The new J curve [107].

It is interesting to note that the famous curve in U (or in J), corresponds to a hormetic profile. It is, therefore, in small doses that alcohol and wine must be consumed, in order to have a beneficial effect on health [105,106] (Figure 10).

Bo showed, in 2017, that the association between alcohol consumption and mortality risk in U.S. adults, follow a J-shaped curve. Compared to abstainers, the all-risk mortality of moderate consumers, were reduced. For heavy alcohol consumption the risk increased significantly [107]. 

Therefore, there are good ways to hope to understand the action of wine polyphenols on human health. First, there is a direct antioxidant effect (scavenger) when wine is absorbed during the meal, after ingestion, polyphenols seems to act as prooxidant. This prooxidant impregnation mechanism, involving the epigenetic expression of endogenous antioxidant enzymes.

## 8. Conclusions

Wine has always been part of the culture of human being, from the east where it was born, to the west where it has taken all its colors and flavors. As far as we can read its footsteps, we can measure its place, both, in the sharing between men and in the benevolent health that fall in the scriptures. Today all scientific studies demonstrate the undeniable part of the quality of nutrition in human health. Wine is unquestionably a health food, on the condition that it is consumed, or is rather savored, in moderation and as a point of conviviality. Current data make it an antioxidant, a repairer of cell damage, a cardiovascular protector, a metabolic and neurological protector [108,109]. Especially wine, in a moderate dose, brings the pleasure of an exchange between guests around a good meal. It is no coincidence that the gastronomic meal of the French was included in the UNESCO’s universal heritage in 2010.

After the study published in the Lancet [110], all alcoholics shouted victory. We are told that wine is an alcohol like the others, and especially that we will die from the first glass of alcohol.

This reminds us of a leaflet from the INCA (The French National Institute of Cancer) in 2009, which has already promised us cancer from the first glass of wine. However, at the same time, Lanzmann-Petithory said, “men who consume mostly wine have a risk of premature mortality from all causes decreased by 25%” [111].

Fortunately, other researchers, but those we talk a little less of, prove other truths. Over ninety studies conducted since 1981 by Dr. Claudia Kawas, 14,000 Californian retirees were followed, the results, as early as 2007, show that men and women who drink alcohol have a lower risk of mortality than the abstainers (those who drank one or two glasses a day saw their mortality reduced by 15%). The study continues and in February 2018, at the American Association for the Advancement of Science conference in Austin, Texas, the results were confirmed—the consumption of two glasses of alcohol per day is associated with a reduction of 18% of the risk of death, compared to abstainers [112]. The study also confirmed that regular exercise, social activities, the regular practice of a hobby and coffee consumption, also increase the life span.

As already mentioned Alvise Cornaro, a Venetian who, after having abused the pleasures of the flesh for 40 years, was given lost by medicine, he was finally able to live to 103 years, after questioning his way of life (frugal food and a little bit of wine). He said: “What we leave after a hearty meal does us more good than we ate” [113].

Wine is the best of anxiolytics. Allowing citizens to relax with a glass of wine, rather than making them feel guilty, could reduce prescriptions for benzodiazepines and antidepressants; while eliminating the side effects of these dangerous drugs. Wine is our panacea. Only misuse or abuse, is dangerous. In conclusion, let us drink fewer, but drink better to live older.
